# Clustering of Major Cardiovascular Risk Factors and the Association with Unhealthy Lifestyles in the Chinese Adult Population

**DOI:** 10.1371/journal.pone.0066780

**Published:** 2013-06-19

**Authors:** Bixia Gao, Luxia Zhang, Haiyan Wang, the China National Survey of Chronic Kidney Disease Working Group

**Affiliations:** Renal Division, Department of Medicine, Peking University First Hospital, Institute of Nephrology, Peking University, Key Laboratory of Renal Disease, Ministry of Health of China, Key Laboratory of Chronic Kidney Disease Prevention and Treatment, Ministry of Education, Beijing, China; University of Sao Paulo, Brazil

## Abstract

**Background:**

Previous studies indicated that lifestyle-related cardiovascular risk factors tend to be clustered in certain individuals. However, population-based studies, especially from developing countries with substantial economic heterogeneity, are extremely limited. Our study provides updated data on the clustering of cardiovascular risk factors, as well as the impact of lifestyle on those factors in China.

**Methods:**

A representative sample of adult population in China was obtained using a multistage, stratified sampling method. We investigated the clustering of four cardiovascular disease (CVD) risk factors (defined as two or more of the following: hypertension, diabetes, dyslipidemia and overweight) and their association with unhealthy lifestyles (habitual drinking, physical inactivity, chronic use of non-steroidal anti-inflammatory drugs (NSAIDs) and a low modified Dietary Approaches to Stop Hypertension (DASH) score).

**Results:**

Among the 46,683 participants enrolled in this study, only 31.1% were free of any pre-defined CVD risk factor. A total of 20,292 subjects had clustering of CVD risk factors, and 83.5% of them were younger than 65 years old. The adjusted prevalence of CVD risk factor clustering was 36.2%, and the prevalence was higher among males than among females (37.9% vs. 34.5%). Habitual drinking, physical inactivity, and chronic use of NSAIDs were positively associated with the clustering of CVD risk factors, with ORs of 1.60 (95% confidence interval [CI] 1.40 to1.85), 1.20 (95%CI 1.11 to 1.30) and 2.17 (95%CI 1.84 to 2.55), respectively. The modified DASH score was inversely associated with the clustering of CVD risk factors, with an OR of 0.73 (95%CI 0.67 to 0.78) for those with modified DASH scores in the top tertile. The lifestyle risk factors were more prominent among participants with low socioeconomic status.

**Conclusion:**

Clustering of CVD risk factors was common in China. Lifestyle modification might be an effective strategy to control CVD risk factors.

## Introduction

Cardiovascular disease (CVD) is the leading cause of mortality and disability worldwide. It is responsible for approximately 30% of global deaths and is an important contributor to the cost of medical care [1]. Hypertension, diabetes, dyslipidemia and overweight are four major CVD risk factors [2]–[4]. Understanding the epidemiological features of those factors is crucial to reducing the growing burden of CVD [5].

Previous studies indicated that CVD risk factors tend to be clustered in certain individuals [6]–. Compelling evidence revealed that those with clustering of CVD risk factors were more likely to develop CVD events, compared with those with a single CVD risk factor [8]. Unhealthy lifestyles, such as physical inactivity and unhealthy diet, have been linked to increased risk of various CVD risk factors [10], [11], and therefore might contribute to the clustering of CVD risk factors. However, population-based studies, especially from developing countries with substantial economic heterogeneity, are extremely limited.

The objective of the present study is to provide current data on the prevalence of clustering of major CVD risk factors in the Chinese adult population, and to identify the relationship between unhealthy lifestyles and clustering of CVD risk factors.

## Materials and Methods

### Study Population

Data from the China National Survey of Chronic Kidney Disease were used for the present analyses, which is a cross-sectional study using a multistage, stratified sampling method to obtain a representative sample of people aged 18 years or older in the Chinese population. Details about the sampling methods were reported elsewhere [12]. In brief, 13 provinces were selected from different geographic regions in China in the first stage of sampling. In the second stage, one urban district and one rural district were selected from each province. In the third stage, three sub-districts (referred to as a “street” and containing approximately 20,000–100,000 households in urban areas; and as a “township” and containing approximately 5,000–30,000 households in rural areas) were selected randomly from the districts. In the fourth stage, five communities were selected randomly from each sub-district. In the final stage, individuals were randomly chosen from each community. A total of 50,550 people were invited to participate in our survey, and 47,204 completed the survey and examination. The response rate was 93%. A total of 521 subjects were excluded from present analyses due to missing data for blood pressure and/or body weight. Finally, a total of 46,683 subjects were included in the analyses.

### Ethics Statement

The ethics committee of Peking University First Hospital approved the study. All of the participants gave written informed consent before data collection.

### Data Collection

All of the on-site screenings were conducted between September 2009 and September 2010. The data were collected in examination centers at local health stations or community clinics in the participants’ residential area. All of the subjects completed a questionnaire documenting their socio-demographic status (e.g., age, sex, and education), personal and family health history (e.g., hypertension, diabetes and CVD), and lifestyle habits with the assistance of medical students, trained general practitioners and nurses.

During the on-site screenings, anthropometric measurements including height, weight, and blood pressure were also collected. Height and weight were measured according to the standard protocol. Height was accurate to 0.1cm and weight was accurate to 0.1 kg. Body mass index (BMI) was calculated as weight in kilograms divided by height in meters squared. Blood pressure was measured using a sphygmomanometer; three measurements were taken at 5min intervals. The mean of the three readings was calculated unless the difference between the readings was greater than 10 mm Hg, in which case the mean of the two closest measurements was used. Serum samples were collected in the morning after an overnight fast and serum creatinine, fasting plasma glucose (FBG) and lipids were measured. Serum creatinine was measured using Jaffe’s kinetic method. FBG was measured enzymatically with a glucose oxidase method. Serum total cholesterol (TC), low-density lipoprotein cholesterol (LDL-C), high-density lipoprotein cholesterol (HDL-C), and triglycerides (TG) were measured with commercially available reagents. The laboratories used a timed-endpoint colorimetric method to measure LDL-C and HDL-C. All of the blood samples were analyzed at the central laboratory in each province. All of the study laboratories successfully completed a standardization and certification program.

All of the study investigators and staff members completed a training program to learn the methods and procedures of the study. A manual of procedures was distributed, and detailed instructions on the administration of the questionnaires, the blood pressure and anthropometric measurements and the collection and processing of biological specimens were provided during the training program.

### Assessment Criteria

We defined four major CVD risk factors: hypertension, diabetes, dyslipidemia and overweight. Clustering of CVD risk factors was defined as presence of at least two major CVD risk factors in one individual. Hypertension was defined as a systolic blood pressure (BP) ≥140 mm Hg and/or a diastolic BP≥90 mm Hg, or the use of antihypertensive medication in the past two weeks regardless of the BP. Diabetes was defined as fasting plasma glucose of 7.0 mmol/L or more or the use of hypoglycemic agents or a self-reported history of diabetes. Dyslipidemia was defined by the presence of at least one of the following: serum TC level ≥5.2 mmol/L, TG level ≥1.7 mmol/L, LDL-C level ≥3.4 mmol/L, and HDL-C level <1.0 mmol/L [13]. Overweight was defined as a BMI ≥24 kg/m^2^ [14].

Four lifestyle risk factors were identified through questionnaire: habitual drinking (defined as drinking everyday), leisure-time physical inactivity (defined as exercising <3.5 hours per week), chronic use of non-steroidal anti-inflammatory drugs (NSAIDS; including phenacetin-containing analgesic mixtures [Somedon and APC] and ibuprofen; chronic use was defined as use at least twice per week for at least 2 months), and a low modified Dietary Approaches to Stop Hypertension (DASH) score. The modified DASH score was calculated from a semi-quantitative questionnaire about intake of fruits, vegetables, legumes, dairy, red meats, total fat and salts (all in quintiles). For the intake of fruits, vegetables, legumes and dairy, quintile 1 was assigned 1 point and quintile 5 was assigned 5 points. While reverse scoring was applied for red meats, total fat, and salts. Then the component scores of each food were summed to create the modified DASH score ranging from 7 to 35. Finally, the modified DASH score was categorized into tertiles (tertile3-highest) [15], [16].

### Statistical Analysis

The prevalence estimates and comparisons were weighted to represent the total adult population of China in 2009. Synthesized weights were calculated by combining three weight values: the sampling weight, the non-response weight and the population weight, which has been described in detail elsewhere [12]. The weights were used to adjust for differential selection probabilities, differential response proportions and deviations in the sample compared to the standard population, particularly in terms of gender and age composition.

Household income was presented as the median (with the inter-quartile range) due to high skewness. Categorical variables were presented as prevalence rate (with 95% confidence intervals [CI]), and all prevalence rates were adjusted for synthesized weights. Relevant characteristics were reported and stratified according to presence of CVD risk factors (none, single, and clustering). The adjusted prevalence rates of clustering of CVD risk factors were reported according to age (18–24 years, 25–29 years, 30–34 years, 35–39 years, 40–44 years, 45–49 years, 50–54 years, 55–59 years, 60–64 years, 65–69 years, and ≥70 years) and gender.

The associations between clustering of CVD risk factors and lifestyle risk factors were analyzed using multinomial Logistic regression models. A lifestyle score (one point for one pre-defined lifestyle risk factor) was calculated based on lifestyle risk factors. The score ranged from zero (most healthy) to four (least healthy) points. The age- and sex-adjusted, as well as multivariable adjusted odds ratios with 95%CI were reported. The covariates included in the multivariable logistic regression models were age (10-year intervals), sex, family history of premature diseases (defined as having first-degree relatives with hypertension, diabetes, stroke and/or coronary heart disease, and with the onset of the disease before 55 years of age for men and before 65 years of age for women) and four defined lifestyle risk factors.

Finally, the socio-economic status (SES; education, and tertiles of household income[tertile3-highest] stratified by region of residency) of participants were compared among participants with 0, 1, ≥2 lifestyle score.

The manuscript was drafted by following the Strengthening the Reporting of Observational Studies in Epidemiology (STROBE) checklist for cross-sectional studies. Data entry and management were performed using Epidata software, version 3.1 (Epidata Association, Odense, Denmark). All p values were 2-tailed and a p value <0.05 was considered to be significant. All analyses were performed using SUDAAN software version 10 (Research Triangle Institute, Raleigh, NC, United States) and SAS software version 9.1 (SAS Institute Inc. Cary, NC, United States).

## Results

**Figure 1:**
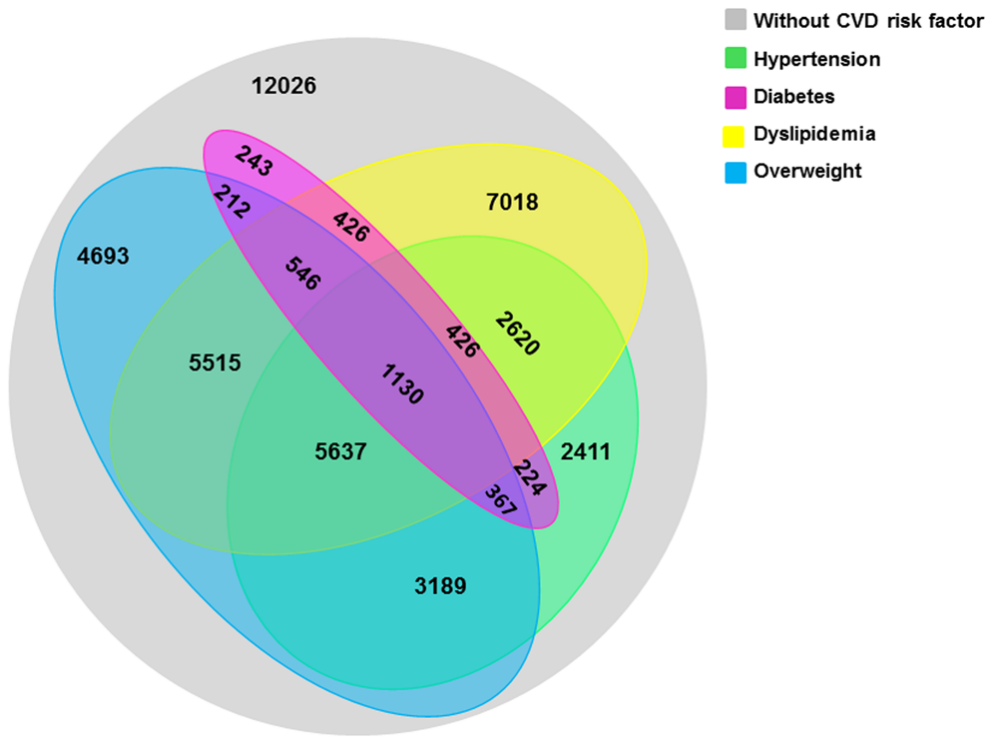
Distribution of four major CVD risk factors in the total sample. Grey represents the population with no defined CVD risk factors, and the other four colors represent the population with four different CVD risk factors. The numbers represent the number of individuals in the population with defined CVD risk factors in the respective regions.

The distribution of the four major CVD risk factors is shown in Figure 1, and the characteristics of the participants according to CVD risk factors are presented in Table 1. Among 46,683 individuals in our study, only 12,026 (adjusted prevalence: 31.1%) were free of pre-defined CVD risk factors, and 14,365 (adjusted prevalence: 32.7%) suffered from a single CVD risk factor (Table 1). A total of 20,292 subjects presented with clustering of CVD risk factors, which represents 58.6% of those with CVD risk factors (Table 1). Furthermore, 15,235 (adjusted prevalence: 83.5%) of participants with clustered CVD risk factors were younger than 65 years (Table 1). The adjusted prevalence of clustering of CVD risk factors was 36.2% (95% confidence interval [CI] 35.4% to 37.1%) (Table 1). Compared with participants without any defined CVD risk factor or with a single major CVD risk factor, participants with the clustering of CVD risk factors were less educated, and were more likely to have a family history of premature diseases (Table 1).

**Table 1:** Characteristics of participants according to CVD risk factors.*

	CVD risk factors			Total	
	None	Single	Cluster		
Number	12 026	14 365	20 292	46 683	
Prevalence (%)	31.1(30.2–31.9)	32.7(31.9–33.6)	36.2(35.4–37.1)		
Age(years)					
18–44	77.0(75.7–78.2)	60.6(59.1–62.0)	42.0(40.6–43.5)		59.0(58.1–59.8)
45–64	18.3(17.2–19.4)	30.0(28.7–31.3)	41.5(40.2–42.9)		30.5(29.8–31.3)
≥65	4.7(4.1–5.3)	9.4(8.7–10.2)	16.4(15.5–17.3)		10.5(10.0–10.9)
Male (%)	47.7(46.0–49.5)	50.7(49.1–52.3)	52.9(51.5–54.3)		50.6(49.6–51.5)
High school education or above (%)	37.8(36.1–39.5)	31.2(29.8–32.7)	25.8(24.6–27.0)		31.3(30.5–32.2)
Have health insurance (%)	90.9(89.9–91.9)	91.7(90.9–92.5)	93.5(92.8–94.1)		92.1(91.6–92.6)
Household income (1000 RMB per year) *	24(9–48)	24(9–48)	24(9–48)		24(9–48)
Family history of premature diseases (%)†	15.2(13.8–16.7)	18.9(17.5–20.3)	25.4(24.0–26.7)		20.1(19.3–20.9)
Lifestyle risk factors					
Habitual drinking (%)	3.1(2.6–3.7)	5.6(5.0–6.3)	8.1(7.3–8.8)		5.7(5.3–6.1)
Leisure-time physical inactivity (%)	82.6(81.3–84.0)	81.7(80.4–82.9)	84.2(83.2–85.2)		82.9(82.2–83.6)
Chronic use of NSAIDs (%)	1.9(1.4–2.5)	4.3(3.6–5.0)	5.0(4.4–5.6)		3.8(3.4–4.2)
Modified DASH score in tertile1 (%)	29.0(27.3–30.7)	33.8(32.2–35.4)	35.3(33.9–36.8)		32.8(31.9–33.8)
Lifestyle score					
0	14.1(12.9–15.3)	14.4(13.2–15.5)	11.5(10.7–12.3)		13.2(12.6–13.9)
1	58.1(56.2–60.0)	51.6(49.9–53.3)	51.1(49.5–52.6)		53.5(52.5–54.5)
≥2	27.8(26.1–29.6)	34.0(32.3–35.7)	37.4(35.9–38.9)		33.3(32.4–34.2)

CVD = cardiovascular disease; RMB = Ren Min Bi; NSAIDS = non-steroidal anti-inflammatory drugs; DASH = dietary approaches to stop hypertension.

The categorical variables are presented as prevalence rate (95% confidence intervals), and all prevalence rates are adjusted for synthesized weights.

* Household income is presented as the median (inter-quartile range) because of substantial skewness.

† First-degree relatives suffered from hypertension, diabetes, stroke and/or coronary heart disease, and the onset was before the age of 55 for men or before the age of 65 for women.

**Figure 2:**
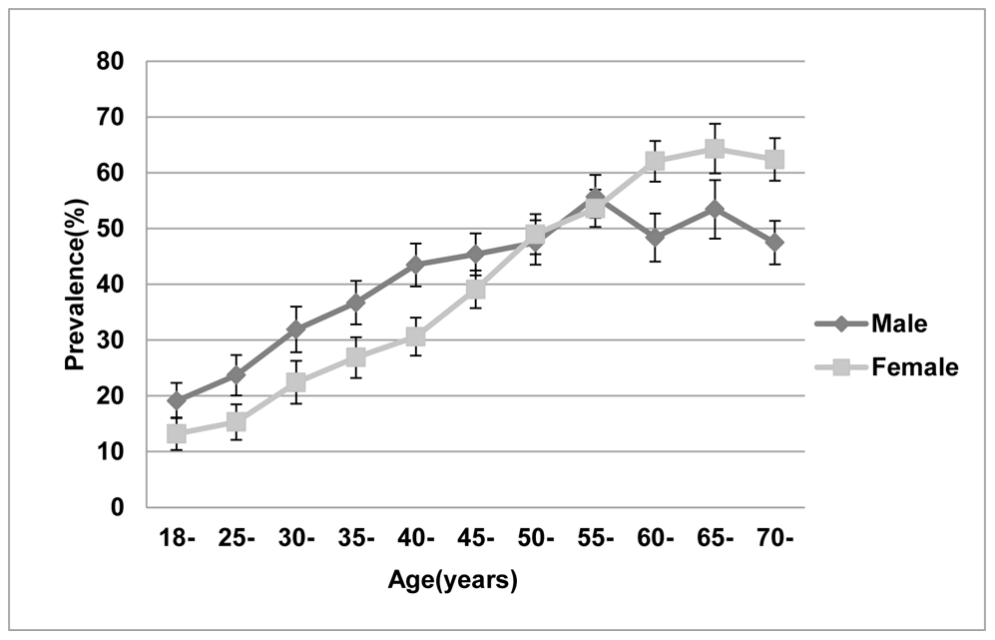
Prevalence of CVD risk factor clustering by age and gender. The prevalence (and 95% confidence intervals) of clustering of CVD risk factors was calculated for various age groups and for both sexes (male or female). All prevalence rates were adjusted for synthesized weights.

The overall prevalence of clustering was higher among males (adjusted prevalence: 37.9%, 95%CI 36.6% to 39.1%) than among females (adjusted prevalence: 34.5%, 95%CI 33.4% to 35.7%), but the prevalence of clustering was higher among females than among males for the participants aged 60 years or older (Figure 2).

The multinomial Logistic regression models revealed that a family history of premature disease was associated with the clustering of CVD risk factors (OR: 1.69; 95%CI 1.52 to 1.88). Multiple lifestyle risk factors, including habitual drinking, physical inactivity, and chronic use of NSAIDs, were positively associated with the clustering of CVD risk factors (OR:1.60 [95%CI: 1.40 to1.85]; OR: 1.20 [95%CI: 1.11 to 1.30]; and OR: 2.17 [95%CI: 1.84 to 2.55], respectively) (Table 2). The modified DASH score was inversely associated with the clustering of CVD risk factors, with an OR of 0.73 (95%CI:0.67 to 0.78) for those with DASH scores in the top tertile. In addition, the OR for clustering increased as the lifestyle score increased; the OR was 1.24 (95%CI: 1.08 to 1.42) for a lifestyle score of 1 and 1.78 (95%CI: 1.55 to 2.03) for a lifestyle score ≥2 (Table 2).

**Table 2:** Clustering of four major CVD risk factors and lifestyle risk factors.*

	Age- and sex- adjusted OR(95%CI)		Fully adjusted OR (95%CI) †	
	Single	Cluster	Single	Cluster
Habitual drinking	1.40(1.21–1.62)	1.78(1.55–2.04)	1.34(1.15–1.55)	1.60(1.40–1.85)
Leisure-time physical inactivity	0.97(0.90–1.04)	1.31(1.22–1.42)	0.90(0.84–0.97)	1.20(1.11–1.30)
Chronic use of NSAIDs	2.19(1.86–2.58)	2.25(1.92–2.65)	2.18(1.85–2.57)	2.17(1.84–2.55)
Modified DASH score				
Tertile1(low)	1.00	1.00	1.00	1.00
Tertile2(middle)	0.88(0.82–0.93)	0.88(0.83–0.94)	0.88(0.83–0.94)	0.91(0.86–0.97)
Tertile3(high)	0.76(0.71–0.82)	0.71(0.66–0.77)	0.75(0.70–0.81)	0.73(0.67–0.78)
Lifestyle score				
0	1.00	1.00	1.00	1.00
1	0.92(0.81–1.04)	1.23(1.07–1.41)	0.92(0.81–1.04)	1.24(1.08–1.42)
≥2	1.25(1.11–1.41)	1.78(1.56–2.03)	1.24(1.09–1.40)	1.78(1.55–2.03)

CVD =  cardiovascular disease; OR = odds ratio; CI = confidence interval; NSAIDS = non-steroidal anti-inflammatory drugs; DASH = dietary approaches to stop hypertension.

* The data are presented as odds ratios (95% confidence intervals); all p<0.001.

† The variables in the fully adjusted models included age, sex, family history of premature diseases and all of the variables in Table 2.

The percentage of lifestyle score of 0 was higher among those with higher education, and among urban residents, compared with their counterparts. On the contrary, the percentage of lifestyle score of 1 or ≥2 was higher among those with lower education, and among rural residents (Table 3). In rural area, the percentage of lifestyle score of 1 or ≥2 was lowest among participants with high tertiles income, while in urban area, the percentage was lowest among participants with low tertiles income (Table 3).

**Table 3:** Relationship between lifestyles and socioeconomic status.*

	Lifestyle score		
	0	1	≥2
Education			
High school or below	28.7(26.5–30.9)	69.5(68.2–70.8)	79.4(78.0–80.9)
> High school	71.3(69.1–3.5)	30.5(29.2–31.8)	20.6(19.1–22.0)
Rural household income tertile1(low)	2.9(2.0–3.8)	26.5(25.2–27.8)	24.8(23.3–26.3)
tertile2(middle)	24.9(22.4–27.5)	44.4(43.0–45.8)	52.6(50.9–54.3)
tertile3(high)	8.4(6.8–9.9)	10.2(9.4–11.0)	9.9(8.9–11.0)
Total	36.2(33.6–38.8)	81.1(80.3–81.9)	87.3(86.6–88.1)
Urban household income tertile1(low)	1.5(1.1–1.8)	1.0(0.8–1.1)	1.6(1.3–1.9)
tertile2(middle)	13.8(12.5–15.1)	7.1(6.7–7.6)	5.8(5.3–6.3)
tertile3(high)	48.5(46.1–50.9)	10.8(10.2–11.4)	5.2(4.7–5.7)
Total	63.8(61.2–66.4)	18.9(18.1–19.7)	12.7(11.9–13.4)

* The data are presented as weighted prevalence rates (95% confidence intervals).

## Discussion

In this representative sample of Chinese adults, we found that over half of those with CVD risk factors presented with a cluster of risk factors. The epidemiological feature of CVD risk factor clustering is valuable for designing targeted intervention strategies, and prevention and intervention strategies should be initiated at younger age. Multiple modifiable lifestyle-related risk factors were associated with the clustering of CVD risk factors. Therefore, comprehensive lifestyle interventions may be an effective strategy for controlling CVD risk factors, especially among those with low SES.

Our study revealed that family history of premature disease was strongly associated with CVD risk factor clustering. In fact, epidemiological studies have demonstrated that CVD risk factors could cluster in twins and among coronary prone family members [17], suggesting that genetic factors might play an important role in the development of CVD risk factors [18]. Whether these CVD risk factors share a common pathogenic gene is still unclear and needs further study.

Our study demonstrated that excessive alcohol consumption, physical inactivity, and chronic use of NSAIDs were positively associated with the clustering of CVD risk factors, while a DASH-style diet was inversely associated with the clustering. This results indicates that modifiable lifestyle factors might influence the development of clusters of CVD risk factors. Observational studies have documented that adhering to a healthy lifestyle in young adulthood is associated with a low CVD risk profile in middle age [19]. In addition, lifestyle interventions (e.g., physical exercise and consuming a low-fat diet) could effectively prevent the development of type 2 diabetes, hypertension and dyslipidemia in high-risk subjects [20]–[22]. Thus, encouraging healthy lifestyles should help to decrease the prevalence of CVD risk factors. Presumably, the government needs to design effective public health policies at the national level, such as advocating healthy dietary pattern, constructing exercise places in community and workplace, and developing strict supervision mechanism to control the abuse of NSAIDs, to modify these unhealthy lifestyles.

In our study, we found that multiple unhealthy lifestyles were more concentrated among those with low education level and among rural residents, indicating that lifestyle modification should be a priority in socioeconomically disadvantaged populations in China. The relationship between SES and health has always been an important public health concern [23]. The data from the 1993 China Health and Nutrition Survey demonstrated that individuals in the high SES group had less healthy lifestyles [24]; however, epidemiological studies in recent years have observed a greater prevalence of unhealthy lifestyles among individuals of lower SES, as seen in our own study [25]. SES might have an impact on access to chronic disease prevention and control knowledge, health care quality, and accessibility of and adherence to medical treatment, and these factors may eventually result in health disparities inequality between various SES levels [26]–[28].

Our study has limitations that deserve mention. Firstly, oral glucose-tolerance test was not performed in our study. Secondly, the laboratory tests were done at central laboratories of 13 study provinces. Therefore, the possibility of variation between laboratories exists. However, all participating laboratories completed a standardization and certification program before the study. Thirdly, the determination of lifestyles were based on self-reported questionnaire. Fourthly, the household income was inquired in 6 categories and then the median of every category were used for calculation, which could affect the precision of measurement. The identical inter-quartile ranges of household income across groups of CVD risk factors might be a reflection of that limitation. Finally, the cross-sectional design limited our ability to make causal inference regarding lifestyles and CVD risk factors.

In conclusion, clustering of CVD risk factors is common in China, and constitute a major challenge to public health. Multiple lifestyle risk factors are responsible for the clustering of CVD risk factors. Therefore, comprehensive interventions to address multiple lifestyle risk factors at the population level might be feasible and cost-effective for controlling the burden of CVD risk factors and clusters of risk factors in China. A systematic large-scale educational effort, especially targeted toward residents with low SES, is also needed.
